# Pattern of Bacterial Pathogens and Their Susceptibility Isolated from Surgical Site Infections at Selected Referral Hospitals, Addis Ababa, Ethiopia

**DOI:** 10.1155/2016/2418902

**Published:** 2016-06-30

**Authors:** Walelign Dessie, Gebru Mulugeta, Surafael Fentaw, Amete Mihret, Mulu Hassen, Engida Abebe

**Affiliations:** ^1^Department of Medical Laboratory Science, College of Health Science, Addis Ababa University, P.O. Box 1176, Addis Ababa, Ethiopia; ^2^Ethiopian Public Health Institute, P.O. Box 1242, Addis Ababa, Ethiopia; ^3^St. Paul's Specialized Hospital Millennium Medical College, P.O. Box 1271, Addis Ababa, Ethiopia

## Abstract

*Background*. The emergence of multidrug resistant bacterial pathogens in hospitals is becoming a challenge for surgeons to treat hospital acquired infections.* Objective*. To determine bacterial pathogens and drug susceptibility isolated from surgical site infections at St. Paul Specialized Hospital Millennium Medical College and Yekatit 12 Referral Hospital Medical College, Addis Ababa, Ethiopia.* Methods*. A cross-sectional study was conducted between October 2013 and March 2014 on 107 surgical site infected patients. Wound specimens were collected using sterile cotton swab and processed as per standard operative procedures in appropriate culture media; and susceptibility testing was done using Kirby-Bauer disc diffusion technique. The data were analyzed by using SPSS version 20.* Result*. From a total of 107 swabs collected, 90 (84.1%) were culture positive and 104 organisms were isolated.* E. coli* (24 (23.1%)) was the most common organism isolated followed by multidrug resistant* Acinetobacter* species (23 (22.1%)). More than 58 (75%) of the Gram negative isolates showed multiple antibiotic resistance (resistance ≥ 5 drugs). Pan-antibiotic resistance was noted among 8 (34.8%)* Acinetobacter* species and 3 (12.5%)* E. coli*. This calls for abstinence from antibiotic abuse.* Conclusion*. Gram negative bacteria were the most important isolates accounting for 76 (73.1%). Ampicillin, amoxicillin, penicillin, cephazoline, and tetracycline showed resistance while gentamicin and ciprofloxacin were relatively effective antimicrobials.

## 1. Introduction

Surgical site infection (SSI) is defined as a proliferation of pathogenic microorganisms which develops in an incision site either within the skin and subcutaneous fat (superficial) and musculofascial layers (deep) or in an organ or cavity, if opened during surgery [1].

Hospital acquired surgical site infections (HAIs) are one of the major health problems throughout the world and are a serious complication affecting hospitalized patients [2, 3]. Pathogens that are able to survive in the hospital environment for long period and resist disinfection are particularly more important for HAIs [2]. SSIs account for a high proportion of the total number of HAIs and have a great impact on patients health care cost, morbidity, and mortality worldwide [3, 4]. SSIs account for 20% to 25% of all hospital acquired infections worldwide [5]. Globally, surgical site infection rates have been reported to range from 2.5% to 41.9% [5]. The risk of acquiring hospital infection on hospitalized patients in relation to surgery is high, since about 77% of death of patients with hospital acquired infections was reported to be related with postoperative infections [3]. The rate of HAIs is markedly higher in many developing countries [2, 3]. The number of surgical patients in developing countries is also increasing but surgical care given to the patients is poor [3].

HAIs are further complicated by an increasing prevalence of multidrug resistant organisms like methicillin resistant* Staphylococcus aureus* (MRSA), methicillin resistant coagulase negative staphylococci (MRCoNS), vancomycin resistant enterococci (VRE) species, multidrug resistance* Escherichia coli, *and* Acinetobacter *spp. [2, 6–8].

The battle between bacteria and their susceptibility to drugs is yet problematic among public, researchers, clinicians, and drug companies who are looking for effective drugs. In addition, SSI by resistant bacteria worsens the condition and it has become serious problem in developing countries like Ethiopia owing to poor infection prevention program, crowding hospital environment, widespread uses of antibiotics, and irrational prescription of antimicrobial agents. Furthermore, recent studies assessing the bacterial isolates of SSIs and their susceptibility pattern in Ethiopia are scarce. Therefore, identification of a microbe and determining susceptibility pattern are beneficial to the patient and assist in selection of chemotherapy to avoid emergence of multidrug resistance organisms in hospital [3, 4]. It is also essential to take appropriate steps to control the spread of infection within the unit. Furthermore, the information gathered helps in planning antibiotic usage policy for SSI. Thus, the aim of this study was to assess bacterial pathogens and drug susceptibility pattern of surgical site infections at selected referral hospitals in Addis Ababa, Ethiopia.

## 2. Materials and Methods

### 2.1. Study Setting and Period

A hospital based cross-sectional study was undertaken at St. Paul's Hospital Millennium Medical College and Yekatit 12 Hospital Medical College in Addis Ababa, Ethiopia, from October 2013 to March 2014. These hospitals are tertiary referral hospitals directly under the Federal Ministry of Health. They is also a teaching hospital for the Medical College and it gives service to the patients under different clinical disciplines which include surgery, orthopedics, obstetrics, gynecology, pediatrics, internal medicine, and ENT.

### 2.2. Study Subjects

Information and clinical sample which are relevant to the study were collected from the study subjects during the study period. A total of 107 patients who developed SSI during the study period were included in the study. All eligible surgical patients were subjected to daily surveillance for the development of infection. This was done according to the clinical criteria for surgical site infection development in CDC SSI classification system (superficial incisional SSI, deep incisional SSI, and organ/space SSI) [1, 9, 10]. Patients who were involuntary to give consent were excluded.

### 2.3. Data and Specimen Collection

Wound swabs were taken from consented surgical site infected patients for microbiological analysis before wound dressing time to avoid skin flora contamination. The wound swab was collected by trained data collectors using sterile cotton swab on a separate sterile test tube or nutrient broth media. Patient specific sociodemographic characteristics and medical histories and all the other required information were collected using the structured questionnaire from the patient medical record and the responsible surgeon, when necessary [10].

### 2.4. Processing of Specimens Culture and Identification

Pus specimens were transported immediately following collection by placing each swab in sterile nutrient broth media to the microbiology laboratory. All the specimens were inoculated onto blood agar, mannitol salt agar, and MacConkey's agar within one hour of collection. The agar plates were incubated at 35–37°C aerobically and examined for the presence of any growth after 24 hours. Those plates showing no growth were incubated for another 24 hours. The isolates were identified by colonial morphology, Gram's stain, and conventional biochemical tests such as catalase, coagulase, oxidase, and mannitol fermentation for Gram positive bacteria and urease, indole, citrate, and sugar utilization tests for Gram negative bacteria [3, 11].

### 2.5. Antimicrobial Susceptibility Testing

Antibiotic susceptibility pattern of the isolates was studied by using Kirby-Bauer technique according to the criteria of the Clinical Laboratory Standards Institute (CLSI) by disc diffusion method. From a pure culture 3–5 pure colonies of bacteria were taken and transferred to a tube containing 5 mL sterile nutrient broth (Oxoid) and they were mixed gently until the turbidity of the suspension become adjusted to a 0.5 McFarland standard. Using sterile cotton swab, the bacteria were seeded evenly over the entire surface of Mueller-Hinton agar (pH 7.2–7.4) (Oxoid). The plates were left at room temperature to dry for 3–5 minutes and antibiotic discs (Oxoid) with the recommended concentrations were placed on the surface of a Muller-Hinton agar plate. Finally, the plates were incubated at 35–37°C for 18–24 hours. Diameters of growth inhibition around the discs were measured and interpreted as sensitive, intermediate, or resistant as per the standard protocol [2, 3, 12]. The following antimicrobial agents were used with their respective concentration. Gentamicin (CN, 10 *μ*g), ciprofloxacin (CIP, 5 *μ*g), and tetracycline (TTC, 30 *μ*g) were used for both Gram positive and Gram negative bacteria. Penicillin (P, 10 units), erythromycin (E, 15 *μ*g), cefoxitin (FOX, 30 *μ*g), and sulfamethoxazole-trimethoprim (SXT, 1.25 *μ*g) were used for Gram positive bacteria while amoxicillin/clavulanic acid (AMC, 20 *μ*g), ampicillin (AMP, 30 *μ*g), chloramphenicol (C, 30 *μ*g), ceftriaxone (CRO, 30 *μ*g), ceftazidime (CAZ, 30 *μ*g), cefuroxime sodium (CXM, 30 *μ*g), cephazoline (KZ, 30 *μ*g), and cefotaxime (CTX, 30 *μ*g) were used for Gram negative bacteria.

### 2.6. Quality Assurance

The reliability of the study findings was guaranteed by implementing quality control (QC) measures throughout the whole processes of the laboratory works. All materials, equipment, and procedures were adequately controlled, and each procedures were aseptically performed. Culture media were tested for sterility and performance. International Control bacteria strains,* E. coli* ATCC 25922,* P. aeruginosa* ATCC 27853, and* S. aureus *ATCC 25923, were included in controlling the tests carried out in this study according to the CLSI. To standardize the inoculums density of bacterial suspension for the susceptibility test, a barium sulfate (BaSO4) turbidity standard, equivalent to a 0.5 McFarland standard, was used [2, 3, 11–13].

### 2.7. Statistical Analysis

The sociodemographic, clinical, and antimicrobial data were obtained from the patient's card and were extracted using a structured questionnaire. The patient's microbiology report was linked to the questionnaire with a code and was documented for each patient on the questionnaire. The information retrieved was used to analyze the rate of surgical site infection and the bacterial isolates and their susceptibility pattern. Data were analyzed using Statistical Package for Social Sciences (SPSS) version 20. Study findings were explained in words, percentage, and tables.

### 2.8. Ethical Considerations

The study was approved after it was ethically reviewed by the research ethical review committee of Department of Medical Laboratory Sciences, Addis Ababa University. Then permission was obtained from the institutional review board of Saint Paul's Hospital Millennium Medical College and Yekatit 12 Referral Hospital Medical College. Written informed consent was obtained from operated patients who developed infection prior to sampling by respective ward nurses. For each confirmed infection by the laboratory analysis, the responsible clinician of the subjects was informed and treatment was started as per the guideline in the hospital. Information obtained at each course of the study was kept confidential.

## 3. Result

A total of 1088 operations were done during the study period. Of these, 107 (9.8%) patients developed surgical site infection. The median age of the study population was 30 years (8–80 years), and the majority (56 (52.3%)) of the study cases were females. Fifty-eight patients (54.2%) were from rural areas, 49 (45.8%) completed primary education, and 33 (30.8%) were farmers. About sixty-two percent of the study population underwent emergency surgery and the most common surgical procedure was laparotomy (34 (31.8%)), followed by debridement (29 (27.1%)). Of the 107 patients studied, 101 (94.4%) received antimicrobial therapy. Twenty-two patients (20.5%) received preoperative antimicrobial therapy, 100 (93.4%) received postoperative antimicrobial therapy, and 6 (5.6%) have not received any antimicrobial therapy (Table 1). There was no significant difference between antimicrobial classes used during the preoperative period and those used during the postoperative period.

### 3.1. Bacterial Isolates

A total of 107 pus specimens were collected and processed during the study period. A total of one hundred and four organisms were isolated from 90 (84.1%) culture positive cases. Seventy-six specimens yielded growth of single organism while two isolates were present in rest of the fourteen cases. The remaining 17 (15.9%) had no bacterial growth. Among the Gram positive isolates,* S. aureus* (19 (18.3%)) was predominant while* E. coli* (23.1%),* Acinetobacter* species (22.1%), and* K. pneumoniae* (9.6%) were the most common isolates of the Gram negative rods. The other isolates were* Pseudomonas*,* Proteus* species,* K. ozaenae*,* Citrobacter*, coagulase negative* Staphylococcus,* and group B streptococci (Table 2).

Both Gram positive and Gram negative bacterial isolates were identified in superficial and deep tissue or organ/space SSI (Table 3).* Acinetobacter *spp. and* S. aureus* were found to be the most prevalent organisms in superficial SSI which account for 13 (26.5%) and 11 (22.4%), respectively, while* E. coli* was the most prevalent organism in deep tissue or organ/space SSI which accounts for 15 (27.3%).

### 3.2. Antimicrobial Susceptibility Pattern

*S. aureus* strains were resistant to penicillin (18 (94.7%)). Cases of resistance to gentamicin and ciprofloxacin were 3 (15.8%) for each and cefoxitin/MRSA were 2 (10.5%). More than 75% of CoNS were resistant to sulfamethoxazole-trimethoprim, cefoxitin, ciprofloxacin, gentamicin, penicillin, and erythromycin. More than 80% of group B streptococci species were susceptible to all of the antibiotics tested for Gram positive bacteria except penicillin and cefoxitin which showed 80% and 60% resistance, respectively (Table 4).

Gram negative rods isolated were considered to be highly resistant to most of the antibiotics tested. Of the isolates, 72 (94.7%), 68 (89.5%), 60 (78.9%), 57 (75%), 56 (73.7%), 50 (65.8%), 38 (50%), and 35 (46.1%) were found to be resistant to ampicillin, cephazoline, cefuroxime sodium, amoxicillin/clavulanic acid, cefotaxime and ceftriaxone (each), ceftazidime, tetracycline, and ciprofloxacin in their respective order, while chloramphenicol (39 (51.3%)), gentamicin (37 (48.7%)), and ciprofloxacin (35 (46.1%)) were relatively effective against Gram negative bacterial isolates (Table 5).

Among the Gram negatives, the isolates of the predominant organism* E. coli* were resistant to tetracycline and cefotaxime (20 (83.3%) each), ampicillin (23 (95.8%)), cephazoline (22 (91.7%)), cefuroxime sodium (21 (87.5%)), ceftriaxone (20 (83.3%)), amoxicillin/clavulanic acid and ceftazidime (17 (70.8%) each), and ciprofloxacin (16 (66.7%)). Chloramphenicol (18 (75%)) was effective against* E. coli*.* Acinetobacter* species also showed high level of resistance to ampicillin, cephazoline, and cefuroxime sodium (100% each), amoxicillin and ceftazidime (19 (82.6%) each), cefotaxime and ceftriaxone (18 (78.3%) each), and chloramphenicol 17 (74%). Tetracycline (60.9%) was relatively effective against* Acinetobacter* species.* Klebsiella* species demonstrated high level of resistance to ampicillin and amoxicillin (10 (100%) each), cephazoline and ceftriaxone (9 (90%) each), ceftazidime (8 (80%)), cefotaxime (7 (70%)), and cefuroxime sodium (6 (60%)). Relatively ciprofloxacin (8 (80%)), tetracycline (7 (70%)), and chloramphenicol and gentamicin (6 (60%) each) were effective against* Klebsiella* species.* P. aeruginosa* demonstrated high level of resistance to ampicillin, amoxicillin, cephazoline, cefuroxime sodium, and cefotaxime (100% each) and ceftriaxone (5 (83.3%)). This organism was susceptible to gentamicin (100%) and ciprofloxacin (2 (66.7%)).* Citrobacter* species isolates were resistant to ampicillin, amoxicillin, and cephazoline (100% each) whereas they were susceptible to ciprofloxacin, ceftriaxone, cefotaxime, gentamicin, and tetracycline (100% each).* Proteus vulgaris* was resistant to ampicillin (100%) and chloramphenicol (3 (75%)), whereas ciprofloxacin, ceftazidime, and gentamicin (100% each) were effective against this species.* P. mirabilis* were susceptible to all the antibiotics tested except tetracycline.

### 3.3. Multidrug Resistance Pattern of Bacterial Isolates

More than 75% of the Gram negative isolates demonstrated evidences of multiple antibiotics resistance (resistance ≥ 5 drugs). Pan-antibiotic resistance was noted among 8 (34.8%)* Acinetobacter* species and 3 (12.5%)* E. coli*. Twenty-two (95.7%) of* Acinetobacter* species were resistant to at least five of the antibiotics tested. Twenty (83.3%) of* E. coli*, 4 (66.7%) of* P. aeruginosa,* and 6 (60%) of* K. pneumoniae* were resistant to more than five of the antibiotics tested. Among the Gram negative isolates, 1 (4.2%)* E. coli* was susceptible to all of the antibiotics tested.

In contrast, 50% of Gram positive isolates were resistant to at least one of the antibiotics tested. Twelve (63.2%), 5 (26.3%), and 1 (5.3%) of the* S. aureus* isolates were found to be resistant to one, two, and at least five of the antibiotics tested, respectively. One isolate was susceptible to all of the antibiotics tested. Half of CoNS were resistant to at least five of the antibiotics (Table 6). Pan-antibiotic resistance was not observed in Gram positive bacteria.

## 4. Discussion

Successful management of patients with bacterial infection depends on early identification of bacterial pathogens and selection of an effective antibiotic against the organism. Antibiotics are one of the pillars of modern medical care and play a major role as both the prophylaxis and treatment of infectious diseases. The issues of their availability, selection, and proper use are of critical importance to the global community [2, 3].

The overall SSI rate (9.8%) was found to be higher than the rates obtained by studies conducted by Amare et al. [3] which were 3.5%, while it is comparable to Amenu et al. [14], Gelaw et al. [2], and Khan et al. [9] who reported prevalence of 11.4%, 7.3%, and 9.3%, respectively, in surgical patients. Other studies also reported similar findings in different countries at different time [15, 16].

The current findings showed 73.1% and 28.9% of Gram negative and Gram positive bacteria, respectively, which is comparable with a study done by Gelaw et al. [2] on surgical hospital acquired infections which reported 69.4% Gram negative bacteria and 30.6% Gram positive bacteria. Predominance of Gram negative organisms in SSI is also reported in some other recent studies [2, 3, 8, 15, 17].

However, other previous studies showed a higher proportion of Gram positive organisms, specially* S. aureus*, associated with SSI in different countries [7, 13, 18–23]. According to CDC,* S. aureus*, CoNS, and* E. coli* were the most prevalent organisms associated with surgical wound infections [24]. This difference in the pattern of distribution of bacterial isolates in different setups may be due to diversity of the study population and local antimicrobial use pattern which results in the emergence of pathogens that have the potential to resist antibiotics used currently. Another reason for the predominance of Gram negative organisms may be the fact that most of the infected patients in our study had undergone abdominal surgery and Gram negatives are predominantly reported to be involved in intra-abdominal procedures [15].

The profiles of bacterial isolates highly associated with the infections in this study were* E. coli*,* S. aureus*,* Klebsiella *species,* P. aeruginosa*, and* Proteus* species which is consistent with previous studies in Ethiopia, Vietnam, Nigeria, and India [3, 4, 7, 25].

In contrast to previous studies in Ethiopia, multidrug resistant* Acinetobacter *species was isolated from 22.1% of the patients which is comparable to a study conducted by Bibi et al. (25.3%) [15]. This finding is again reported in previous studies in Pakistan, Vietnam, and Brazil [13, 17, 26]. This is due to its environmental desiccation survival for weeks, a characteristic that promotes transmission through common hospital sources of contamination [27].

From the total isolated bacteria, 94.4% were isolated from patients who received antibiotic therapy before and after surgery which is in agreement with similar study reported from Gondar and Addis Ababa [3, 28]. The present study showed relatively frequent isolation among patients who received antibiotic prophylaxis and the most commonly prescribed drug for prophylaxis was ceftriaxone alone or in combination with other antibiotics such as gentamicin, chloramphenicol, and amoxicillin. This shows some antibiotics alone or in combination require periodic evaluation and the establishment of antibiotics policy for prophylaxis and treatment guidelines in the Ethiopian setting.

Antimicrobial sensitivity testing of the isolates showed higher rates of multidrug resistant (MDR) strains of these organisms in SSI. More than 18 (90%) isolates of* S. aureus* showed resistance to penicillin. This finding is in agreement with previous studies [3, 14]. Above 75% of CoNS were resistant to sulfamethoxazole-trimethoprim, cefoxitin, ciprofloxacin, gentamicin, penicillin, and erythromycin which is comparable to a study conducted in Gondar [3]. Almost all group B streptococci species were susceptible to all of the antibiotics tested for Gram positive bacteria, but penicillin and cefoxitin showed resistance.

Majority of Gram negative bacteria showed very high resistance to ampicillin, cephazoline, ceftriaxone, cefuroxime sodium, amoxicillin/clavulanic acid, cefotaxime, ceftazidime, and tetracycline which is in agreement with different studies worldwide [3, 4, 6, 13–15, 19]. The high rate of bacterial resistance against ceftriaxone, ampicillin, and amoxicillin is likely due to frequent use of these antibiotics both within hospital and outside.

*E. coli, Acinetobacter *species,* K. pneumoniae, P. aeruginosa,* and* P. vulgaris* showed higher resistance to amoxicillin, ampicillin, cefotaxime, cefuroxime sodium, and ceftriaxone (*β*-lactam antibiotics). This high resistance of organisms to the most commonly used antibiotics (*β*-lactam antibiotics) was reported from many studies [2, 3, 6, 14, 22] in Ethiopia. Similarly, a study in Pakistan and India reported the high resistance of* Acinetobacter* species and* P. aeruginosa* isolated from surgical wounds [15, 25].

Pan-antibiotic resistance was noted among 8 (34.8%) of* Acinetobacter* species. Resistance is due to mechanisms that are expressed frequently in hospital acquired strains of* Acinetobacter* including *β*-lactamases, alterations in cell-wall channels (porins), and efflux pumps [27].

The resistance rates for* E. coli* isolate were as follows: tetracycline and cefotaxime: 20 (83.3%) each, ampicillin: 23 (95.8%), cephazoline: 22 (91.7%), ceftriaxone and cefuroxime sodium: 21 (87.5%) each, amoxicillin and ceftazidime 17 (70.8%) each, and ciprofloxacin: 16 (66.7%). Chloramphenicol was active against* E. coli*.

Gentamicin and ciprofloxacin were relatively effective antibiotics against Gram negative organisms associated with SSI which is in agreement with previous study conducted in Ethiopia [17]. The frequency of single as well as multiple drug resistance was alarmingly high. This might be a sign of inappropriate use of antimicrobials and lack of performing antimicrobial susceptibility test.

Due to limited laboratory facilities and expertise, we were unable to investigate anaerobic bacterial and fungal agents. Therefore, future studies should be conducted to include these organisms that require special media and environment for growth.

## 5. Conclusion and Recommendation

Gram negative organisms with multiple drug resistance were commonly associated with postoperative surgical site infection. Rational antimicrobial use and continuing surveillance of bacterial antimicrobial sensitivity tests at local level are necessary to reduce emergence and spread of resistant bacterial isolates. The practice of aseptic technique during and after surgery should be the primary support rather than overreliance on antibiotics to reduce emergence and spread of resistant pathogens. It is also recommended that gentamicin and ciprofloxacin should be used in preference to ceftriaxone and amoxicillin for treatment of postoperative surgical site infections. Pan-antibiotic resistant* Acinetobacter* species needs special implementation and monitoring of antibiotics.

## Figures and Tables

**Table 1:** Sociodemographic and clinical characteristics of study subjects (*n* = 107).

Characteristics	Frequency	Percentage
*Sex*		
Female	56	52.3
Male	51	47.7
*Age group*		
<10	2	1.9
10–20	18	16.8
21–30	36	33.6
31–40	23	21.5
41–50	9	8.4
>50	19	17.8
*Residence*		
Urban	49	45.8
Rural	58	54.2
*Educational status*		
Illiterate	29	27.1
Primary	49	45.8
Secondary	14	13.1
College/university and above	15	14
*Occupations*		
Farmer	33	30.8
Employer	17	15.9
Student	13	12.1
Daily wage laborer	14	13.1
Housewife	16	15
Merchant	6	5.6
Jobless	5	4.7
Driver	3	2.8
*Case type*		
Emergency	66	61.7
Elective	41	38.3
*Surgical procedure*		
Laparotomy	34	31.8
Debridement	29	27.1
Appendectomy	3	2.8
Drainage	9	8.4
Incision	5	4.7
Cesarean section	14	13.1
Colostomy	8	7.5
Lithotomy	3	2.8
Mastectomy	2	1.9
*SSI class*		
Superficial	45	42.1
Deep tissue or organ/space	62	57.9
*Antimicrobial therapy received *		
Preoperative	22	20.5
Postoperative	100	93.4
Not at all	6	5.6

**Table 2:** Bacterial pathogens isolated from postoperative surgical site infection.

Bacterial isolate	Frequency	Percent
*Gram positive *	*28*	*26.9*
*S. aureus*	19	18.3
CoNS^*∗*^	4	3.8
Group B streptococci spp.	5	4.8

*Gram negative*	*76*	*73.1*
*E. coli*	24	23.1
*Acinetobacter *spp.	23	22.1
*K. pneumoniae*	10	9.6
*K. ozaenae*	3	2.9
*P. aeruginosa*	6	5.8
*P. vulgaris*	6	5.8
*P. mirabilis*	1	0.9
*Morganella* spp.	1	0.9
*Citrobacter *spp.	2	1.9

*Total*	*104*	*100*

^*∗*^CoNS: coagulase negative staphylococci; spp.: species.

**Table 3:** Bacterial distribution based on SSI class.

Bacterial isolate	Superficial SSI: number (%)	Deep tissue or organ/space SSI: number (%)	Total
*S. aureus*	11 (22.4)	8 (14.5)	19 (18.3)
CoNS^*∗*^	4 (8.2)	0 (0)	4 (3.8)
Group B streptococci spp.	2 (4.1)	3 (5.5)	5 (4.8)
*E. coli*	9 (18.4)	15 (27.3)	24 (23.1)
*Acinetobacter *spp.	13 (26.5)	10 (18.2)	23 (22.1)
*K. pneumoniae*	3 (6.1)	7 (12.7)	10 (9.9)
*K. ozaenae*	1 (0.2)	2 (3.6)	3 (2.9)
*P. aeruginosa*	2 (4.1)	4 (7.3)	6 (5.8)
*P. vulgaris*	3 (6.1)	3 (5.5)	6 (5.8)
*P. mirabilis*	0 (0)	1 (1.8)	1 (0.9)
*Morganella* spp.	0 (0)	1 (1.8)	1 (0.9)
*Citrobacter *spp.	1 (0.2)	1 (1.8)	2 (1.9)

Total	49	55	**104**

^*∗*^CoNS: coagulase negative staphylococci; spp.: species.

**Table 4:** Antibiotic susceptibility pattern of Gram positive bacterial isolates in postoperative surgical site infected patients in selected referral hospitals of Addis Ababa.

Bacterial isolate	Pattern	Antimicrobial agents						
		CN	CIP	TE	PE	SXT	FOX	E
*S. aureus*	S	16 (84.2)	16	15 (78.9)	1 (5.3)	15 (78.9)	17 (89.5)	15 (78.9)
	R	3 (15.8)	3 (15.8)	4 (21.1)	18 (94.7)	4 (21.1)	2 (10.5)	4 (21.1)

CoNS	S	1 (25)	0 (0)	3 (75)	1 (25)	1 (25)	0 (0)	1 (25)
	R	3 (75)	4 (100)	1 (25)	3 (75)	3 (75)	4 (100)	3 (75)

Group B streptococci spp.	S	5 (100)	—	5 (100)	1 (20)	5 (100)	2 (40)	4 (80)
	R	0 (0)		0 (0)	4 (80)	0 (0)	3 (60)	1 (20)

E = erythromycin, SXT = sulfamethoxazole-trimethoprim, FOX = cefoxitin, P = penicillin.

**Table 5:** Antibiotic susceptibility pattern of Gram negative bacterial isolates in postoperative surgical site infected patients in selected referral hospitals of Addis Ababa.

Bacterial isolate	Pat	Antimicrobial agents, *n* (%)										
		CTX	C	CN	AMP	AMC	CRO	CAZ	KZ	CIP	CXM	TE
*E. coli*	S	4 (16.7)	18 (75)	11 (45.8)	1 (4.2)	3 (12.5)	4 (16.7)	4 (16.7)	2 (8.3)	8 (33.3)	3 (12.5)	4 (16.7)
	I	0 (0)	0 (0)	0 (0)	0 (0)	4 (16.7)	0 (0)	1 (4.1)	0 (0)	0 (0)	0 (0)	0 (0)
	R	20 (83.3)	6 (25)	13 (54.2)	23 (95.8)	17 (70.8)	20 (83.3)	19 (79.2)	22 (91.7)	16 (66.7)	21 (87.5)	20 (83.3)

*Acinetobacter* spp.	S	5 (21.7)	6 (26)	6 (26.1)	0 (0)	3 (13)	5 (21.7)	4 (17.4)	0 (0)	8 (34.8)	0 (0)	14 (60.9)
	I	0 (0)	0 (0)	4 (17.4)	0 (0)	1 (4.3)	0 (0)	0 (0)	0 (0)	0 (0)	0 (0)	0 (0)
	R	18 (78.3)	17 (74)	13 (56.5)	23 (100)	19 (82.6)	18 (78.3)	19 (82.6)	23 (100)	15 (65.2)	23 (100)	9 (39.1)

*K. pneumoniae*	S	1 (10)	6 (60)	6 (60)	0 (0)	0 (0)	1 (0)	2 (20)	1 (10)	8 (80)	4 (40)	7 (70)
	R	7 (70)	4 (40)	4 (40)	10 (100)	10 (100)	9 (90)	8 (80)	9 (90)	2 (20)	6 (60)	3 (30)

*K. ozaenae*	S	0 (0)	3 (100)	2 (66.7)	0 (0)	0 (0)	0 (0)	0 (0)	0 (0)	3 (100)	0 (0)	2 (66.7)
	R	3 (100)	0 (0)	1 (33.3)	3 (100)	3 (100)	3 (100)	3 (100)	3 (100)	0 (0)	3 (100)	1 (33.3)

*P. aeruginosa*	S	0 (0)	1 (16.7)	5 (83.3)	0 (0)	0 (0)	1 (16.7)	4 (66.7)	0 (0)	4 (66.7)	0 (0)	3 (50)
	I	0 (0)	2 (33.3)	1 (16.7)	0 (0)	0 (0)	0 (0)	0 (0)	0 (0)	0 (0)	0 (0)	0 (0)
	R	6 (100)	3 (50)	0 (0)	6 (100)	6 (100)	5 (83.3)	2 (33.3)	6 (100)	2 (33.3)	6 (100)	3 (50)

*P. vulgaris*	S	3 (75)	1 (25)	4 (100)	0 (0)	2 (50)	3 (75)	4 (100)	2 (50)	4 (100)	3 (75)	0 (0)
	R	1 (25)	3 (75)	0 (0)	4 (100)	2 (50)	1 (25)	0 (0)	2 (50)	0 (0)	1 (25)	4 (100)

*P. mirabilis*	S	1 (100)	1 (100)	1 (100)	1 (100)	1 (100)	1 (100)	1 (100)	1 (100)	—	—	0 (0)
	R	0 (0)	0 (0)	0 (0)	0 (0)	0 (0)	0 (0)	0 (0)	0 (0)			1 (100)

*Morganella* spp.	S	1 (100)	1 (100)	—	0 (0)	0 (0)	1 (100)	1 (100)	0 (0)	—	—	0 (0)
	R	0 (0)	0 (0)		1 (100)	1 (100)	0 (0)	0 (0)	1 (100)			1 (100)

*Citrobacter* spp.	S	2 (100)	2 (100)	2 (100)	0 (0)	0 (0)	2 (100)	1 (50)	0 (0)	—	—	2 (100)
	R	0 (0)	0 (0)	0 (0)	2 (100)	2 (100)	0 (0)	1 (50)	2 (100)			0 (0)

S = sensitive, I = intermediate, R = resistant, TE = tetracycline, CIP = ciprofloxacin, AMP = ampicillin, AMC = amoxicillin/clavulanic acid, CTX = cefotaxime, CAZ = ceftazidime, KZ = cephazoline, CXM = cefuroxime sodium, C = chloramphenicol, CN = gentamicin, CRO = ceftriaxone.

**Table 6:** Multidrug resistance pattern of bacterial isolates.

Bacterial isolate	Total number (%)	Number (%) of antimicrobial patterns					
		*R*_0_	*R*_1_	*R*_2_	*R*_3_	*R*_4_	≥*R*_5_
*Gram negative*	76 (73.1)	1 (1.3)	1 (1.3)	3 (3.9)	6 (7.6)	7 (9.2)	58 (76.3)
*E. coli*	24 (23.1)	1 (4.2)	0 (0)	0 (0)	1 (4.2)	2 (8.3)	20 (83.3)
*Acinetobacter*	23 (22.1)	0 (0)	0 (0)	0 (0)	1 (4.3)	0 (0)	22 (95.7)
*K. pneumoniae*	10 (9.6)	0 (0)	0 (0)	2 (20)	1 (10)	1 (10)	6 (60)
*K. ozaenae*	3 (2.9)	0 (0)	0 (0)	0 (0)	0 (0)	0 (0)	3 (100)
*P. aeruginosa*	6 (5.8)	0 (0)	0 (0)	0 (0)	1 (16.7)	1 (16.7)	4 (66.7)
*P. vulgaris*	6 (5.8)	0 (0)	0 (0)	1 (16.7)	2 (33.3)	1 (16.7)	2 (33.3)
*P. mirabilis*	1 (0.9)	0 (0)	1 (100)	0 (0)	0 (0)	0 (0)	0 (0)
*Morganella* spp.	1 (0.9)	0 (0)	0 (0)	0 (0)	0 (0)	1 (100)	0 (0)
*Citrobacter *spp.	2 (1.9)	0 (0)	0 (0)	0 (0)	0 (0)	1 (50)	1 (50)
*Gram positive*	28 (26.9)	2 (7.1)	14 (50)	7 (25)	1 (3.6)	0 (0)	4 (14.3)
*S. aureus*	19 (18.3)	1 (5.3)	12 (63.2)	5 (26.3)	0 (0)	0 (0)	1 (5.3)
CoNS	4 (3.8)	0 (0)	0 (0)	1 (25)	1 (25)	0 (0)	2 (50)
Group B streptococci spp.	5 (4.8)	1 (20)	2 (40)	1 (20)	1 (20)	0 (0)	0 (0)

*R*_0_: no resistance, *R*_1_: resistance to one drug, *R*_2_: resistance to two drugs, *R*_3_: resistance to three drugs, *R*_4_: resistance to four drugs, ≥*R*_5_: resistance to five or more drugs.

## Abbreviations

CDC: Center for Disease Control and Prevention
CLSI: Clinical Laboratory Standard Institute
ENT: Ear nose and throat
HAIs: Hospital acquired infections
MRSA: Methicillin resistant* Staphylococcus aureus*
QC: Quality control
SOPs: Standard operative procedures
SPSS: Statistical Package for Social Sciences
SSI: Surgical site infection
VRE: Vancomycin Resistant.

## References

[1] Bagnall N. M., Vig S., Trivedi P. (2009). Surgical-site infection. *Surgery*.

[2] Gelaw A., Gebre Selassie S., Tiruneh M., Fentie M. (2013). Antimicrobial susceptibility patterns of bacterial isolates from patients with postoperative surgical site infection, health professionals and environmental samples at a tertiary level hospital, North West Ethiopia. *International Journal of Pharmaceutical Sciences Review and Research*.

[3] Amare B., Abdurrahman Z., Moges B., Ali J. (2011). Postoperative surgical site bacterial infections and drug susceptibility patterns at Gondar University Teaching Hospital, Northwest Ethiopia. *Journal of Bacteriology & Parasitology*.

[4] Thu L. T. A., Sohn A. H., Tien N. P. (2006). Microbiology of surgical site infections and associated antimicrobial use among Vietnamese orthopedic and neurosurgical patients. *Infection Control and Hospital Epidemiology*.

[5] Mawalla B., Mshana S. E., Chalya P. L., Imirzalioglu C., Mahalu W. (2011). Predictors of surgical site infections among patients undergoing major surgery at Bugando Medical Centre in Northwestern Tanzania. *BMC Surgery*.

[6] Mulu W., Kibru G., Beyene G., Damtie M. (2012). Postoperative nosocomial infections and antimicrobial resistance pattern of bacteria isolates among patients admitted at Felege Hiwot Referral Hospital, Bahirdar, Ethiopia. *Ethiopian Journal of Health Sciences*.

[7] Adegoke A., Mvuyo T., Okoh A. I., Steve J. (2010). Studies on multiple antibiotic resistant bacterial isolated from surgical site infection. *Scientific Research and Essays*.

[8] Wolcott R. D., Zischakau Y. A., Dowd S. E. (2009). Bacterial diversity in surgical site infections: not just aerobic cocci any more. *Journal of Wound Care*.

[9] Khan M., Khalil J., Rooh-ul-Muqim (2011). Rate and risk factors for surgical site infection at a tertiary care facility in Peshawar, Pakistan. *Journal of Ayub Medical College, Abbottabad*.

[10] Reichman D. E., Greenberg J. A. (2009). Reducing surgical site infections: a review. *Reviews in Obstetrics & Gynecology*.

[11] Cheesbrough M. (2001). *District Laboratory Practice in Tropical Countries*.

[12] Reddy B. R. (2012). Management of culture negative surgical site infections in India. *Medical & Allied Sciences*.

[13] Mahmood A. (2000). Bacteriology of surgical site infections and antibiotic susceptibility pattern of the isolates at a tertiary care hospital in Karachi. *Journal of the Pakistan Medical Association*.

[14] Amenu D., Belachew T., Araya F. (2011). Surgical site infection rate and risk factors among obstetric cases of Jimma University Specialized Hospital, Southwest Ethiopia. *Ethiopian Journal of Health Sciences*.

[15] Bibi S., Channa G. A., Siddiqui T. R., Ahmed W. (2012). Pattern of bacterial pathogens in postoperative wounds and their sensitivity patterns in Karachi, Pakistan. *Journal of Surgery Pakistan*.

[16] Suchitra Joyce B., Lakshmidevi N. (2009). Surgical site infections: assessing risk factors, outcomes and antimicrobial sensitivity patterns. *African Journal of Microbiology Research*.

[17] Dantas S. R. P. E., Kuboyama R. H., Mazzali M., Moretti M. L. (2006). Nosocomial infections in renal transplant patients: risk factors and treatment implications associated with urinary tract and surgical site infections. *Journal of Hospital Infection*.

[18] Onche I., Adedeji O. (2010). Microbiology of post-operative wound infection in implant surgery. *Nigerian Journal of Surgical Research*.

[19] Shriyan A., Sheetal R., Nayak N. (2010). Aerobic micro-organisms in post-operative wound infections and their antimicrobial susceptibility patterns. *Journal of Clinical and Diagnostic Research*.

[20] Sani R. A., Garba S. A., Oyewole O. A., Ibrahim A. (2012). Antibiotic resistance profile of gram positive bacteria isolated from wound infections in Minna, Bida, Kontagora and Suleja Area of Niger State. *Journal of Health Sciences*.

[21] Wassef M. A., Hussein A., Abdul Rahman E. M., El-Sherif R. H. (2012). A prospective surveillance of surgical site infections: study for efficacy of preoperative antibiotic prophylaxis in Egypt. *African Journal of Microbiology Research*.

[22] Mama M., Abdissa A., Sewunet T. (2014). Antimicrobial susceptibility pattern of bacterial isolates from wound infection and their sensitivity to alternative topical agents at Jimma University Specialized Hospital, South-West Ethiopia. *Annals of Clinical Microbiology and Antimicrobials*.

[23] Dessalegn L., Shimelis T., Tadesse E., Gebre-Selassie S. (2014). Aerobic bacterial isolates from post-surgical wound and their antimicrobial susceptibility pattern: a hospital based cross-sectional study. *E3 Journal of Medical Research*.

[24] National Nosocomial Infections Surveillance (NNIS) System (1996). National Nosocomial Infections Surveillance (NNIS) report, data summary from October 1986–April 1996, issued May 1996. *American Journal of Infection Control*.

[25] Sikka A., Mann J. K., Deep, Vashist M. G., Chaudhary U., Deep A. (2012). Prevalence and antibiotic sensitivity pattern of bacteria isolated from nosocomial infections in a surgical ward. *Indian Journal of Clinical Practice*.

[26] Sohn A. H., Parvez F. M., Vu T. (2002). Prevalence of surgical-site infections and patterns of antimicrobial use in a large tertiary-care hospital in Ho Chi Minh City, Vietnam. *Infection Control and Hospital Epidemiology*.

[27] Munoz-Price L. S., Weinstein R. A. (2008). Acinetobacter infection. *The New England Journal of Medicine*.

[28] Abula T., Kedir M. (2004). The pattern of antibiotic usage in surgical in-patients of a teaching hospital, northwest Ethiopia. *Ethiopian Journal of Health Development*.

